# Shear Stress Alterations Activate BMP4/pSMAD5 Signaling and Induce Endothelial Mesenchymal Transition in Varicose Veins

**DOI:** 10.3390/cells10123563

**Published:** 2021-12-17

**Authors:** Karthika Chandran Latha, Ahalya Sreekumar, Vyshna Beena, Binil Raj S.S., RaviKumar B. Lakkappa, Ravi Kalyani, Radhakrishnan Nair, Saligrama Ramegowda Kalpana, Chandrasekharan C. Kartha, Sumi Surendran

**Affiliations:** 1 Cardiovascular Diseases and Diabetes Biology, Rajiv Gandhi Centre for Biotechnology, Thiruvananthapuram 695014, India; karthikac@rgcb.res.in (K.C.L.); ahalyas@rgcb.res.in (A.S.); vyshnabeena000@gmail.com (V.B.); 2 College of Pharmaceutical Sciences, Medical College, Thiruvananthapuram 695011, India; tobinil@gmail.com; 3 Vascular Surgery, Kempegowda Institute of Medical Sciences, Bangalore 560070, India; drravikumarvascularsurg@gmail.com; 4 Sri Jayadeva Institute for Cardiovascular Sciences & Research, Bangalore 560069, India; ravicardiac@gmail.com (R.K.); kalpanasr@gmail.com (S.R.K.); 5 St. Thomas Institute of Research on Venous Diseases, Changanassery 686104, India; dr.arkay@gmail.com; 6 Department of Neurology, Amrita Institute of Medical Sciences, Amrita Vishwa Vidyapeetham, Kochi 682041, India; cckartha@gmail.com

**Keywords:** varicose veins, shear stress, endothelial cells, EndMT, TGFβ, BMP

## Abstract

Chronic venous diseases, including varicose veins, are characterized by hemodynamic disturbances due to valve defects, venous insufficiency, and orthostatism. Veins are physiologically low shear stress systems, and how altered hemodynamics drives focal endothelial dysfunction and causes venous remodeling is unknown. Here we demonstrate the occurrence of endothelial to mesenchymal transition (EndMT) in human varicose veins. Moreover, the BMP4-pSMAD5 pathway was robustly upregulated in varicose veins. In vitro flow-based assays using human vein, endothelial cells cultured in microfluidic chambers show that even minimal disturbances in shear stress as may occur in early stages of venous insufficiency induce BMP4-pSMAD5-based phenotype switching. Furthermore, low shear stress at uniform laminar pattern does not induce EndMT in venous endothelial cells. Targeting the BMP4-pSMAD5 pathway with small molecule inhibitor LDN193189 reduced SNAI1/2 expression in venous endothelial cells exposed to disturbed flow. TGFβ inhibitor SB505124 was less efficient in inhibiting EndMT in venous endothelial cells exposed to disturbed flow. We conclude that disturbed shear stress, even in the absence of any oscillatory flow, induces EndMT in varicose veins *via* activation of BMP4/pSMAD5-SNAI1/2 signaling. The present findings serve as a rationale for the possible use of small molecular mechanotherapeutics in the management of varicose veins.

## 1. Introduction

The global prevalence of chronic venous diseases, especially varicose veins, remains very high [1]. Curative therapy for varicose veins targeted at causal mechanisms remains elusive as the disease etiopathogenesis is unclear. Valvular incompetence and primary wall changes in superficial saphenous veins are the major pathologic features of varicose veins in the lower extremities of the body [2,3]. These alterations result in blood reflux, venous hypertension, dysfunctional saphenous veins, and associated complications.

The low pressure in the veins and risk-aggravating factors such as orthostatic lifestyle increases the venous filling pressure and lead to hemodynamic disturbances. Disturbed flow can occur naturally in certain regions of the venous system such as tributaries, vein bifurcations, and venous valve sinuses [4,5]. Altered flow because of blood reflux through incompetent valves is also seen in patients having polymorphisms in genes such as the *FoxC2,* which code for valve maintenance [6]. Earlier studies have reported that shear stress in the range of 5–6 dyn/cm^2^ affects endothelial function [7,8,9]. Shear stress in veins is in the range of 1–6 dyn/cm^2^ and is inversely proportional to the vessel diameter [10,11]. The low shear stress under physiological conditions could be protective for venous endothelial cells.

Earlier studies related to atherogenesis have indicated that disturbed shear stress induces endothelial mesenchymal transition (EndMT) [12] in arteries. EndMT is associated with uncontrolled proliferation of smooth muscle cells (SMCs) and vascular remodeling in several diseases, including atherosclerosis [13,14]. Hitherto, the EndMT process has not yet been examined in varicose veins.

In the study reported here, we have investigated whether the disturbed flow can cause EndMT in veins. Signaling pathways underlying EndMT in varicose veins were also explored.

## 2. Materials and Methods

### 2.1. Study Subjects

Varicose veins were collected from 39 patients (29 men and 10 women) with chronic venous diseases (C3) who underwent vein stripping at Kempegowda Institute of Medical Science, Bangalore. The median age of patients was 39 years (range 21–68). Healthy saphenous veins were collected from 44 subjects who underwent coronary artery bypass grafting (38 men and 6 women) at Sri Jayadeva Institute of Cardiovascular Sciences and Research, Bangalore. The median age of control subjects was 55 years (range 36–73). The study was performed following the Declaration of Helsinki and was approved by the Human Ethics Committee at Rajiv Gandhi Centre for Biotechnology. All subjects provided their consent for inclusion before they participated in the study. Inclusion and exclusion criteria were evaluated by a vascular surgeon. Patients with chronic venous diseases were recruited by a vascular surgeon after physical examinations and duplex ultrasound scanning; venous reflux time greater than 1 s was considered for diagnosis. The exclusion criteria were varicosities secondary to neoplasms, patients who had prior vascular surgery of lower limbs, deep vein thrombosis, and comorbidities.

### 2.2. Endothelial Cell Culture and Flow Exposure

Human umbilical vein endothelial cells (HUVEC) were cultured in an endothelial growth medium supplemented with 10% FBS and 1% penicillin-streptomycin cocktail. The cells were maintained at 37 °C in 95% humidified air with 5% CO_2_. Endothelial cells were characterized based on von Willebrand factor (vWF) expression [15] and uptake of acetylated low-density lipoprotein (Ac-LDL) (Invitrogen, USA) [16].

For flow exposure-based experiments, HUVECs were seeded on flow chamber μ slides (Ibidi-Integrated BioDiagnostics, Germany) and grown to confluency. Two types of IBIDI μ slides were used. μ–Slide I 0.4 Luer ibiTreat was used to ensure unidirectional laminar flow mimicking normal venous flow, and μ–slide Y-shaped ibiTreat was used to mimic non-uniform laminar flow mimicking minimal disturbance as seen during early flow pattern in varicose veins. Once cells attained confluency, culture media was allowed to pass through cells using the IBIDI pump system to attain venous shear stress of 6 dyn/cm^2^ [11,17] on cells for a period of 24 and 48 h. The pressure was gradually increased from 2 to 6 dyn/cm^2^ during the initial few h. For static experiments, cells were seeded on μ slide, and shear stress was not applied. The flow system was kept in a humidified atmosphere containing 5% CO_2_. The working mechanism of the IBIDI perfusion system is detailed in Supplementary Figure S1. The flow rates and shear stress were managed by IBIDI pump control v1.5.4 software.

### 2.3. RNA Extraction, cDNA Synthesis, and Quantitative Real-Time PCR

Total RNA was extracted from varicose veins (*n* = 39) and healthy veins (*n* = 44), as well as cells exposed to various flow regimes using the Trizol method (Thermo Fisher Scientific, Carlsbad, CA, USA), according to the manufacturer’s protocol. RNA concentration and purity were measured by a nanodrop-1000 spectrophotometer (Thermo Fisher Scientific, USA). RNA (500 ng) from each sample was reverse-transcribed using oligodT, dNTPs, and M-MLV reverse transcriptase (Promega, Madison, WI, USA). Reaction conditions were: 70 °C for 5 min, 42 °C for 1 h, and 95 °C for 5 min. Quantitative real-time PCR (qRT-PCR) was performed to analyze the expression profiles of genes using the SYBR Green PCR master mix (Applied Biosystems, Columbus, USA) in an ABI Prism 7900HT sequence detection system. The reactions were performed in triplicate in a 96-well plate and by following temperature steps: initial denaturation step at 95 °C for 10 min, with 40 cycles consisting of 95 °C for 15 s, 60 °C for 1 min, and 72 °C for 30 s. mRNA expression was normalized with the expression of endogenous control, *GAPDH*. The dCT method was used to analyze the expression level of each gene, and the expression data are presented as fold change (2^–^^ΔΔCt^). Gene expression in cells exposed to parallel and disturbed flow was normalized to the expression levels in the static culture. The specificity of the amplicon is ascertained by a single peak in the qRT-PCR dissociation curve. Primers sequences and their specific annealing temperature are listed in Supplementary Table S1.

### 2.4. Histology and Immunohistochemistry

Tissue sections (4 μm) were cut from the formalin-fixed, paraffin-embedded varicose vein samples (*n* = 27) and control saphenous veins (*n* = 25). Deparaffinization was performed with xylene and rehydration of sections using graded alcohol series. Hematoxylin and eosin staining of varicose and control veins were performed as described earlier [18].

For immunohistochemistry, the deparaffinized and rehydrated sections were washed in distilled water and incubated in citrate buffer at 95 °C for 25 min for antigen epitope retrieval. Immunostaining was performed using SS Polymer-HRP IHC detection system/ DAB (BioGenex, Fremont, CA, USA). Briefly, endogenous peroxidase was blocked with hydrogen peroxide treatment for 20 min followed by non-specific protein blocking by 5% bovine serum albumin (BSA) (Sigma-Aldrich, St. Louis, MO, USA) for 30 min. Tissue sections were incubated with diluted primary antibodies overnight at 4 °C. Slides were washed 3 times with Tris-buffered saline (TBS) prior to the addition of horseradish peroxidase (HRP)–conjugated secondary antibodies. Negative controls were treated identically but without the primary antibody. Details of primary and secondary antibodies used and their working dilutions are given in Supplementary Table S2. The signals were visualized using 3,3-Diaminobenzidine (DAB) followed by nuclear staining with hematoxylin. Tissue sections were washed, dehydrated in ascending grades of isopropanol, cleared in xylene, mounted in dibutyl-phthalate xylene, and observed under a light microscope (Nikon, Tokyo, Japan). The intensity of the DAB signals in each immunohistochemical image was quantified by ImageJ software. Seven random fields per slide were selected for H score analysis.

### 2.5. Western Blotting

Proteins were extracted from 31 varicose, and 34 control veins (100 µg) using RIPA buffer with protease inhibitor cocktails described earlier [6]. Protein concentrations were determined with the Bradford reagent as per manufactures instructions (Bio-Rad, Hercules, CA, USA). Total proteins were resolved on a 10% SDS-PAGE and transferred onto a nitrocellulose membrane (Bio-Rad, Hercules, CA, USA). Membranes were blocked with 5% BSA in TBS for 1 h to reduce the non-specific binding of proteins, and membranes were incubated with primary antibodies (Supplementary Table S2) at 4 °C overnight. After rinsing thrice with TBS containing 0.1% Tween-20 (TBST), the membrane was incubated with HRP-conjugated secondary antibodies for 1 h at 37 °C in TBST. The chemiluminescence signal was detected using enhanced chemiluminescence (ECL) substrate (Bio-Rad, USA), and chemiluminescence measurements were performed using the Chemi Doc imaging system. Image J software was used for densitometry of immunoreactive bands.

### 2.6. Immunofluorescence Analysis

For the visualization of EndMT-associated proteins, cells were exposed to static, laminar, and disturbed venous shear stress for 24 h. After exposure to flow conditions, cells were washed with PBS, fixed with 4% formaldehyde for 20 min, and were permeabilized with 0.1% Triton X-100 for 10 min. Cells were blocked with 3% BSA (Sigma-Aldrich, USA) in phosphate-buffered saline (PBS) for 1 h at room temperature and then incubated with diluted primary antibodies (Supplementary Table S2) overnight at 4 °C. Slides were washed with PBS and cell samples were exposed to host-specific secondary antibodies for 1 h at room temperature. The absence of primary antibodies was considered as assay negative control. Nuclear staining was performed using Hoechst 33342 (Sigma-Aldrich, USA). Cells were mounted in fluorochrome, examined, and images were captured by confocal microscope (Nikon, Japan). Quantification of the fluorescence signal was performed with Nikon NIS Elements.

### 2.7. Inhibitor Assays

In experiments to prevent disturbed shear stress-induced EndMT pathway, endothelial cells were exposed to disturbed flow in the presence of small molecule inhibitors of TGβ1, i.e., 500 nM SB505124 or BMP4 inhibitor LDN193189 (Sigma-Aldrich, USA), at a concentration of 1 μM for 24 h. The concentrations of inhibitors were selected based on earlier studies [19,20]. Cell viability at these concentrations was checked before the flow-based experiments using MTT assay (Sigma-Aldrich, USA), as described earlier [21].

### 2.8. Statistical Analysis

Data were analyzed, and graphs were prepared using GraphPad Prism version 8 (San Diego, CA, USA). mRNA expression from patient specimens was presented as scatter plots. Densitometry and H score results were presented as bar graphs, and comparisons between disease and healthy controls were measured using one-way ANOVA. In all cell culture experiments, data were expressed as mean ± standard deviation (SD). mRNA expression levels and mean fluorescence intensity of confocal images were also represented by bar graphs. Error bars represent mean ± SD. A *p*-value < 0.05 was generally considered statistically significant (* *p* < 0.05, ** *p* < 0.01, *** *p* < 0.001, **** *p* < 0.0001). Results from cell culture were confirmed by at least three or more biological repeats.

## 3. Results

### 3.1. Neointima of Varicose Veins Express High Levels of Mesenchymal Markers

Previously, we had shown that there is an augmented expression of Dll4, ephrin B2, Hey2, and vimentin in varicose veins compared to controls [6,18]. The higher expression of vimentin and SMC proliferation observed in varicose veins led us to hypothesize that arterialization of saphenous veins does occur during varicose vein pathogenesis. Histological analysis of varicose veins from patients demonstrated thickened vein walls in comparison to control veins (Supplementary Figure S2). Varicose veins had thick intima with ‘neointima’ formation. Circular and longitudinally arranged stacks of SMCs were clustered in tunica media compared to the simple longitudinal arrangement of SMCs in control veins.

**Figure 1:**
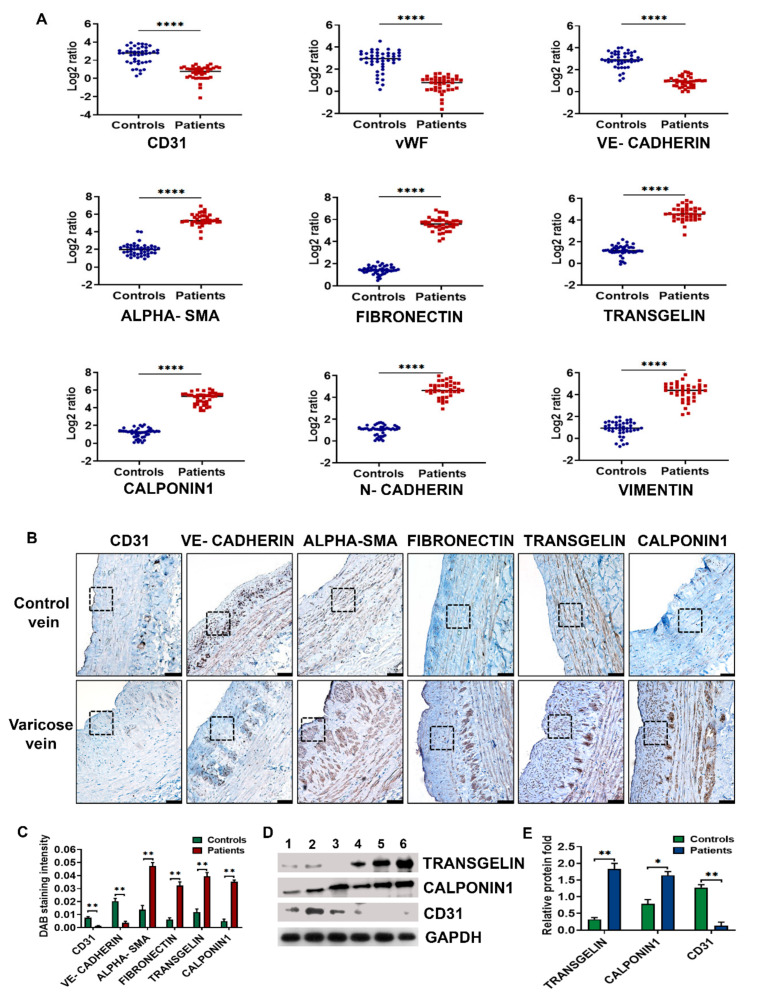
Differential expression of endothelial and mesenchymal markers in varicose veins. (**A**) Scatter plots represent the mRNA fold changes of endothelial markers such as CD31, vWF, VE-cadherin, and mesenchymal α-SMA, fibronectin, transgelin, calponin1, N-cadherin, and vimentin in 39 human varicose veins and 44 control saphenous veins. GAPDH was used as the endogenous control for mRNA fold analysis. Horizontal lines in scatter plots indicate median values. (**B**) Immunohistochemical staining shows the reduced expression of endothelial markers CD31 and VE-cadherin and overexpression of mesenchymal α-SMA, transgelin, fibronectin, and calponin1 in varicose veins compared to healthy saphenous veins. The area enclosed by dotted lines especially shows the differential expression of endothelial and mesenchymal markers in the neointima of varicose veins. In the control vein, the dotted box indicates the protein expression in the intima (CD31, VE-cadherin) or media (α-SMA, transgelin, fibronectin, and calponin1). (Scale bar 100 µM, magnification 20×). (**C**) Bar graph showing semiquantitative H score analysis of endothelial and mesenchymal markers in seven random microscopic fields of immunostaining in 27 varicose and 25 control veins. (**D**) Representative western blots showing the expression levels of transgelin, calponin1, CD31, and loading control GAPDH in healthy saphenous veins (lanes 1–3) and human varicose veins (lanes 4–6). (**E**) Densitometry of immunoblots from 31 varicose and 34 healthy veins indicated significant upregulation of calponin1 and transgelin in varicose veins, with low CD31expression compared to healthy veins. Values are means ± SD (standard deviation) * indicates *p* < 0.05 vs. healthy subjects, ** *p* < 0.01 and **** *p* < 0.0001.

To investigate whether there is EndMT-like phenotype switching, endothelial and mesenchymal markers as well as EndMT-associated transcriptional factors were examined in 39 human varicose veins and 44 control saphenous veins by qRT-PCR. Figure 1A illustrates the low expression of endothelial-specific *CD31, vWF*, VE-cadherin (*CDH1*) in RNA isolated from varicose veins compared to their expressions in control veins. The mRNA transcripts of mesenchymal markers such as α-SMA (*ACTA2*), transgelin (*SM22α*), calponin1 (*CNN1*), fibronectin (*FN1*), vimentin (*VIM*), and N-cadherin (*CDH2*) were significantly elevated in varicose veins.

Expressions of CD31, calponin1, and transgelin proteins in varicose and control veins were further assessed by Western blot, with GAPDH as the loading control (Figure 1D). Densitometry analysis of immunoblots (Figure 1E) indicated significant upregulation of calponin1 and transgelin in varicose veins, with low CD31expression. The results were consistent with the qRT-PCR findings.

Histology of varicose veins revealed a larger neointimal area compared to control saphenous veins (Supplementary Figure S2, Figure 1B). *Tunica media* was thickened, and bundles of SMCs were seen in varicose veins. There was an incomplete endothelial coverage of CD31 and VE-cadherin immunostaining in varicose veins (Figure 1B). VE-cadherin was downregulated in varicose veins by 5.5 folds compared to control tissues. Immunostaining of CD31 was too weak in varicose veins compared to control veins in which CD31 was expressed along the luminal endothelial lining of the intima. In 22 out of 27 varicose veins (81.48%), the endothelium was almostdenuded possibly during the stripping process used for sample collection. Mesenchymal markers such as α-SMA, transgelin, fibronectin, and calponin1 were overexpressed in varicose veins compared to control by 3.42-, 3.3-, 5.25-, and 6.92-fold, respectively. These cytoplasmic proteins were expressed in the neointima and media of varicose veins compared to medial longitudinal bundles of SMCs in healthy veins. Compared to other mesenchymal markers, calponin1 expression was much less in control veins. Figure 1C depicts the semiquantitative differential expression of endothelial and mesenchymal markers in varicose and control veins using H Score analysis. The results of the digital H Score were concordant with those using immunohistochemistry (IHC) images.

### 3.2. BMP4-pSMAD5-SNAI1/2 Signaling Is Upregulated in Varicose Veins

**Figure 2:**
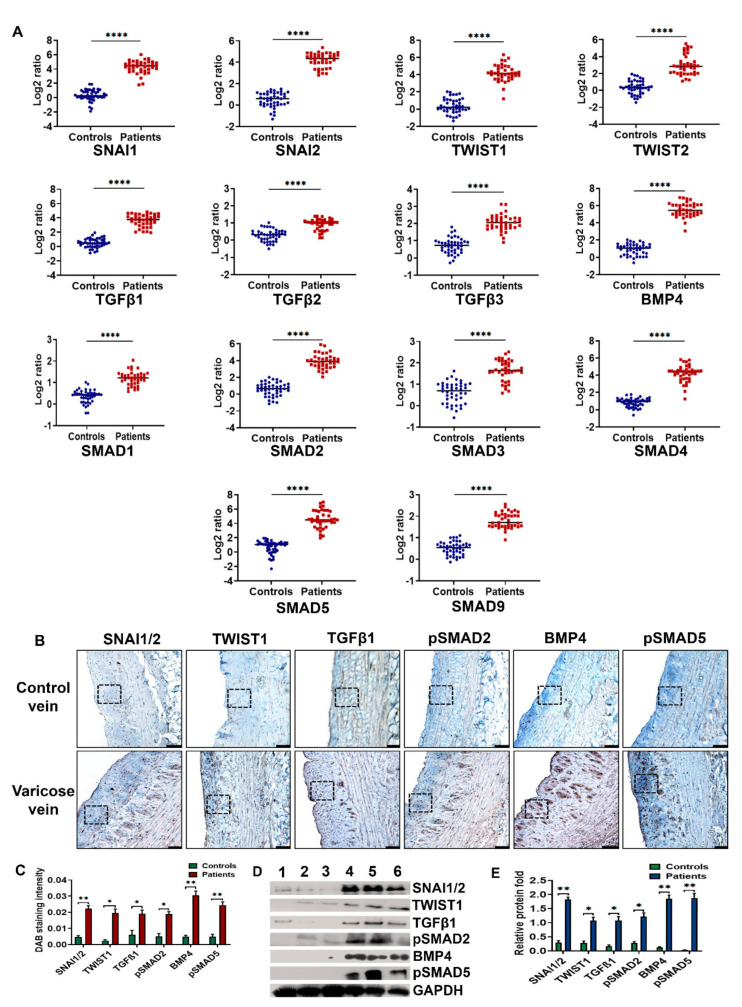
Expression profile of EndMT markers in varicose veins. (**A**) Scatter plots of mRNA fold changes of EndMT-associated genes such as *SNAI1, SNAI2, TWIST1, TWIST2, TGFβ1-3, BMP4, SMAD1-5,* and *SMAD9* in 39 human varicose and 44 control saphenous veins. *GAPDH* was taken as the endogenous control for quantification. Horizontal lines in scatter plots indicate median values. (**B**) Representative photomicrographs of immunostaining illustrate that SNAI1/2, TWIST1, TGFβ1, BMP4, pSMAD2, and pSMAD5 proteins were overexpressed in varicose compared to healthy saphenous veins. The areas enclosed by dotted lines show the differential expression of EndMT markers in the neointima of varicose veins. In the control vein, the dotted box indicates the protein expression in intima. (Scale bar 100 µM, magnification 20×). (**C**) Semiquantitative H score analysis shows overexpression of SNAI1/2, TWIST1, TGFβ1, BMP4, pSMAD2, and pSMAD5 proteins in varicose veins, which was further substantiated with (**D**) Western blot assays performed using total protein extracted from healthy veins (lanes 1–3) and human varicose veins (lanes 4–6). (**E**) Bar graph demonstrating densitometry analysis of immunoblots from 31 varicose veins compared to 34 healthy veins indicated significant upregulation of EndMT markers in varicose veins. Values are means ± SD (standard deviation) * indicates *p* < 0.05 vs. healthy subjects, ** *p* < 0.01 and **** *p* < 0.0001.

To determine the regulatory pathways of EndMT signaling in varicose veins, mRNA transcript levels of key molecules related to TGFβ and BMP pathways were analyzed in veins. We screened RNAs from all of 39 varicose and 44 control veins for *SNAI1, SNAI2, TWIST1, TWIST2, TGFβ1-3, BMP4, SMAD1-5,* and *SMAD9* using qRT-PCR. As shown in Figure 2A, the median values of mRNA expressions of *SNAI1, SNAI2,* and *TWIST1* were observed to be 4.27-, 4.20-, and 4.18-fold, respectively, higher in varicose veins than healthy veins. *TWIST2* expression was not as high as *TWIST1,* but the difference was statistically significant (2.96-fold, *p* < 0.0001 vs. control). *TGFβ1-3* expression in varicose veins was also higher compared to controls. *TGFβ1* expression (3.63-fold) in varicose veins was higher than *TGFβ*2 and *TGFβ3* isoforms (0.93- and 2.04-fold, respectively). Increase in the expression of *BMP4* 5.5-folds higher in varicose veins than in control veins. All SMADs were significantly upregulated in varicose veins, especially *SMAD2* involved in TGFβ signaling*, SMAD5,* and *SMAD9* associated with the BMP pathway as well as co-*SMAD4*.

Western blotting experiments confirmed the overexpression of SNAI1/2, TWIST1, TGFβ1, phospho-SMAD2 (pSMAD2), BMP4, and phospho-SMAD5 (pSMAD5) proteins in varicose veins (Figure 2D). GAPDH was used as the loading control. Densitometry analysis of immunoblots indicated statistically significant upregulation of TGFβ1 and BMP4 pathways-associated EndMT in varicose veins (Figure 2E).

We studied the expression of SNAI1/2, TWIST1, TGFβ1, pSMAD2, BMP4, and pSMAD5 proteins in varicose veins and healthy veins (Figure 2B). SNAI1/2 and TWIST1 variably stained nuclei and cytoplasm of cells in neointima as well as media in varicose veins. SNAI1/2 and TWIST1 staining were negligible in healthy veins. TGFβ1 and BMP4 induce EndMT through the translocation of phosphorylatedSMAD2/3 and SMAD1/5/9, respectively, to the nucleus and thereby express SMAD target genes. Hence TGFβ1, BMP4, pSMAD2, and pSMAD5 expression in veins were examined. TGFβ1 and pSMAD2 were weakly expressed by SMCs in healthy saphenous veins. The overexpression of TGFβ1 and pSMAD2 were noticeable in the neointima, media, and adventitia of 15 out of 27 (55.56%) varicose veins; their expressions were less compared to the intense cytoplasmic expression of BMP4 and pSMAD5. BMP4 and pSMAD5 expressions were intense in 25 out of 27 (92.59%) varicose veins. In two samples, BMP4/pSMAD5 staining was at par with TGFβ1 and pSMAD2. H scores analysis illustrated in Figure 2C revealed that BMP4 and pSMAD5 staining were highly intense in varicose veins compared to TGFβ1 and pSMAD2. The finding indicates an active EndMT process associated with neointimal hyperplasia and venous remodeling in varicose veins.

### 3.3. Disturbed Venous Flow Induces EndMT in Venous Endothelial Cells

Mechanosignaling studies in atherosclerosis report the expression of EndMT markers in endothelial cells in response to low shear stress [7,8,9]. The venous system is a physiologically low shear system. Hence, we hypothesized that alterations in flow rather than its magnitude contribute to the pathological features in vasculature.

To understand the fluid flow-induced differential expression of EndMT genes as we observed in vein samples from patients with varicose veins, we exposed cultured HUVEC to uniform laminar flow (parallel flow) and non-uniform disturbed flow for 24 h and 48 h using an IBIDI pump system. Before the flow-based studies, structural and functional characterization of HUVEC was performed by analyzing the expression of vWF and uptake of Dil-Ac-LDL (Supplementary Figure S3). Shear stress of 6 dyn/cm^2^, comparable to the average level of shear stress found in small veins, was applied [11,17].

**Figure 3:**
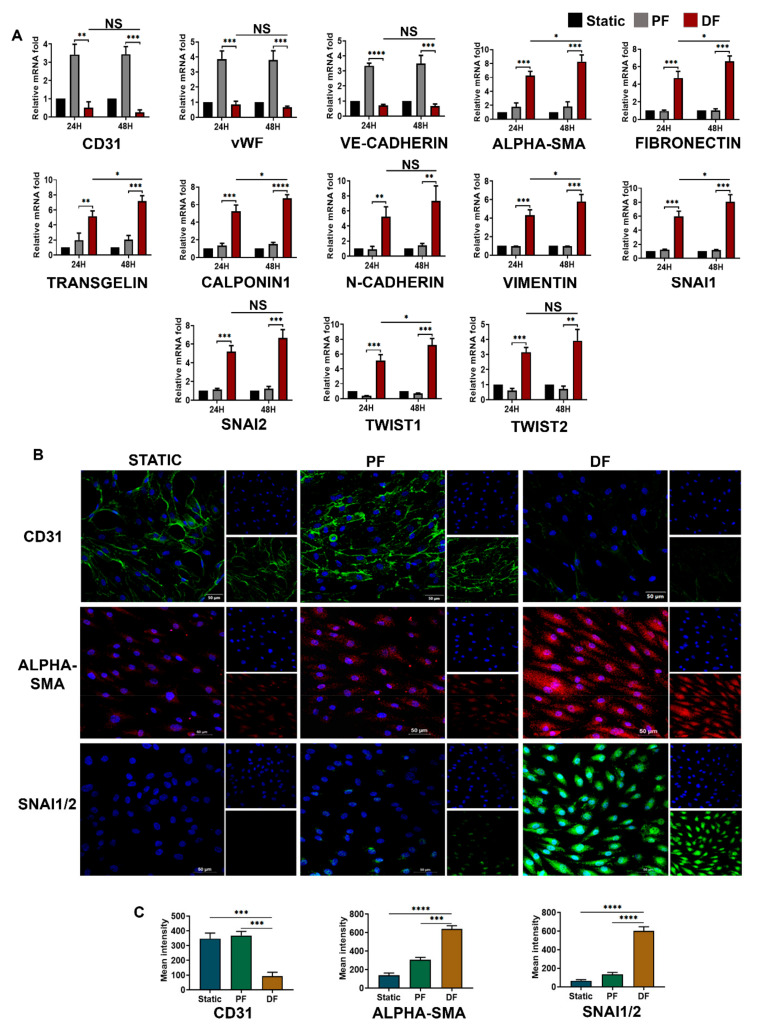
EndMT activation by disturbed fluid flow in venous endothelial cells. (**A**) Time-dependent (24 and 48 h) mRNA-level expression of EndMT-associated transcriptional factors, endothelial markers, and mesenchymal markers upon exposure of HUVEC to disturbed flow (*n* = 3). EndMT becomes prominent as endothelial cells are exposed to disturbed flow for longer periods. The loss of endothelial markers and gain of mesenchymal markers was significant after exposure to disturbed flow for 48 h. mRNA fold values in parallel and disturbed flow were calculated relative to the static control. All data were normalized with GAPDH expression and are given as relative to static control. (**B**) HUVECS exposed to disturbed flow at 6 dyn/cm^2^ for 24 h resulted in an intermittent pattern of membrane-bound CD31 expression with an intense α-SMA staining compared to parallel uniform flow and static control conditions. SNAI1/2 transcription factors expression was augmented in the cells exposed to disturbed flow. SNAI1/2 expression was mostly confined to the cell nucleus, but several cells had both nuclear and cytoplasmic SNAI1/2 localization too (scale bar 50 µM, magnification 40×). (**C**) Mean fluorescence intensity was plotted as the average fluorescence intensity ± standard deviation (SD) of five microscopic fields per flow condition and from three biological replicates. * represents *p* < 0.05, ** *p* < 0.01, *** *p* < 0.001, and **** *p* < 0.0001 vs. respective static or parallel uniform shear-treated groups.

qRT-PCR analysis confirmed a decreased expression of endothelial markers such as CD31, vWF, and VE-cadherin in cells exposed to disturbed flow (Figure 3A). We also observed that mRNA transcript of mesenchymal markers such as αSMA (6.26-folds), transgelin (5.17-folds), N-cadherin (5.26-folds), fibronectin (4.72-folds), calponin1 (5.24-folds), and vimentin (4.32-folds) were augmented in HUVECs exposed to disturbed flow compared to cells exposed to static flow conditions for 24 h. Similarly, transcriptional factors active in EndMT such as *SNAI1, SNAI2, TWIST1,* and *TWIST2* were significantly induced by the disturbed flow. For instance, *SNAI1* was expressed approximately 5.995-fold in disturbed flow compared to 1.046-fold in static culture and 1.203-fold in cells exposed to parallel uniform flow.

Time-point analysis (Figure 3A) indicated that endothelial mesenchymal phenotype switching becomes prominent as exposure time increases. The loss of CD31 and VE-cadherin was significant after exposure to disturbed flow for 48 h. Disturbed flow prevents the parallel flow-induced increase in CD31 and other endothelial proteins. Downregulation of CD31 may have a role in reduced endothelial integrity and suggest the initiation of endothelial dysfunction in early venous remodeling. The expressions of mesenchymal markers also increased significantly with the duration of exposure of cells to disturbed flow.

To verify the changes at the protein level, immunofluorescence staining of cells for endothelial CD31, mesenchymal α-SMA, and SNAI1/2 transcriptional factors were performed (Figure 3B). In HUVECs under static flow, the membrane-bound expression of CD31 was prominent. Cells exposed to parallel uniform venous shear stress had higher expression of CD31 in their membranes. However, the protein-level expression of CD31 in parallel flow was not as higher as the mRNA-level upregulation. There was progressive loss of CD31 under disturbed flow. We also found that levels of α-SMA cytoskeletal protein were significantly elevated in cells exposed to disturbed flow, suggesting that HUVECs acquired a mesenchymal phenotype in 24 h. Staining for EndMT-inducing transcription factors, SNAI1/2 was intense in cells exposed to disturbed non-uniform flow. Notably, SNAI1/2 localized to the nucleus of endothelial cells in response to disturbed flow, suggesting that it is active under flow disturbances. A peak fluorescence intensity was found in α-SMA and SNAI1/2-stained cells, which were exposed to disturbed flow, compared to other flow conditions (Figure 3C). The parallel flow was shown to slightly induce SNAI1/2 and α-SMA expression in HUVEC, possibly due to the low flow magnitude we selected. This low flow shear stress in the presence of minute non-uniform disturbance significantly increased the α-SMA and SNAI1/2 expression. We concluded that non-uniform shear stress promotes mesenchymal phenotype transition in venous endothelial cells. Interestingly, no such switch was observed in cells exposed to uniform low shear stress.

### 3.4. TGFβ1-pSMAD2 Signaling in Venous Endothelial Cells Exposed to Disturbed Flow

**Figure 4:**
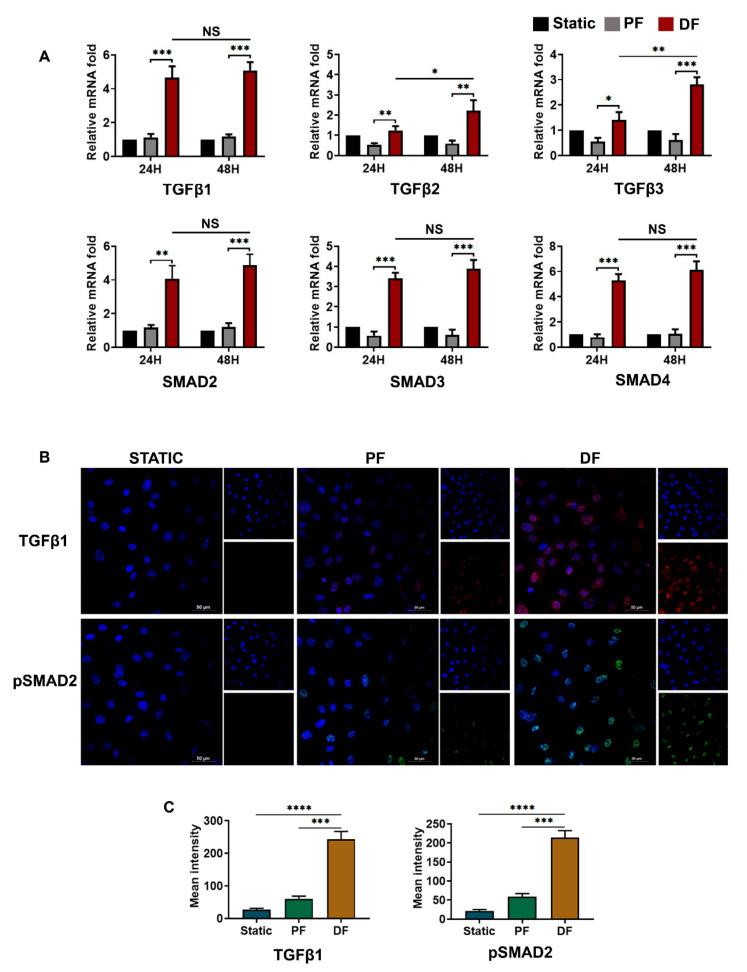
TGFβ activation by disturbed fluid flow in venous endothelial cells. (**A**) Time-dependent (24 and 48 h) mRNA expression of *TGFβ1, TGFβ2,* and *TGFβ3* ligands as well as their signaling partners *SMAD2, SMAD3,* and *co-SMAD4* upon exposure of HUVEC to disturbed flow (*n* = 3). *TGFβ1* and *SMADs* showed significant differences in expression between endothelial cells exposed to disturbed flow and parallel uniform flow as early as 24 h of exposure. The expression of *TGFβ2* and *TGFβ3* was significant after exposure to disturbed flow for 48 h. mRNA fold values were calculated relative to static control. All data were normalized with GAPDH expression and are given as relative to static control. (**B**) HUVECS exposed to disturbed flow at 6 dyn/cm^2^ for 24 h, showed overexpression of TGFβ1 and pSMAD2 (scale bar 50 µM, magnification 40×). (**C**) Mean fluorescence intensity was plotted as the average fluorescence intensity ± SD of five fields per flow condition and from three biological replicates. Analysis of five fields indicated an active TGFβ1- pSMAD2 pathway in the cells exposed to disturbed flow. PF indicates parallel uniform shear stress, and DF represents disturbed shear stress without any oscillatory flow. * indicates *p* < 0.05, ** *p* < 0.01, *** *p* < 0.001, and **** *p* < 0.0001 vs. respective static or parallel uniform shear-treated groups.

We next analyzed the expression of *TGFβ1-3*and *SMAD2-4* in cells exposed to various flow conditions (Figure 4). We observed a significant difference in *TGFβ1* expression between endothelial cells exposed to disturbed flow (4.67-fold vs. static) and parallel uniform flow (1.13-fold vs. static) (*p* < 0.001). *TGFβ2* and *TGFβ3* were also expressed more in cells exposed to disturbed flow (*p* < 0.05), but their expressions were prominent only on longer exposure periods. *SMAD2/3* and co-*SMAD4* were elevated at mRNA levels in cells exposed to disturbed flow when compared to static and parallel flow conditions (Figure 4A).

Figure 4B illustrates the expression of TGFβ1 and pSMAD2in cells exposed to various flow conditions. TGFβ1 and pSMAD2 were overexpressed in response to disturbed flow conditions. (static vs. disturbed flow: *p* < 0.0001) (Figure 4C). Nuclear translocation of active pSMAD2 was confirmed by immunofluorescence staining.

### 3.5. BMP4-pSMAD5 Is Significantly Expressed in Venous Endothelial Cells Exposed to Disturbed Flow

**Figure 5:**
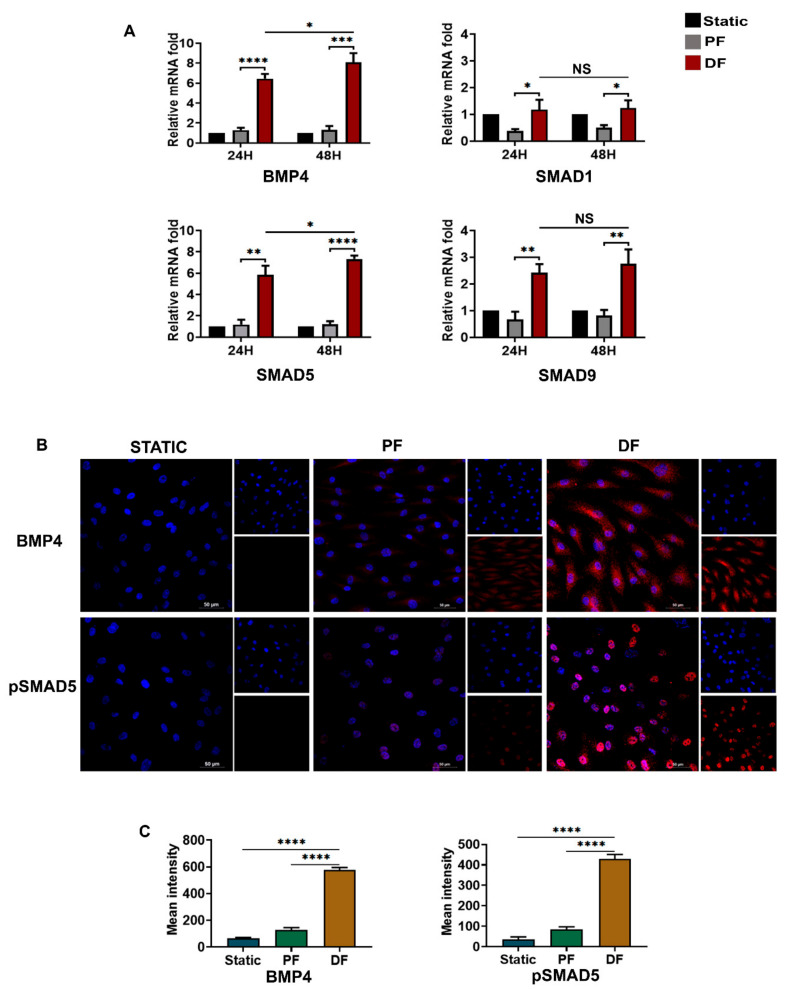
Activation of BMP4-pSMAD5 signaling by disturbed fluid flow in venous endothelial cells. (**A**) Time-dependent (24 and 48 h) mRNA-level expression of *BMP4* ligand as well as its effectors *SMAD1, SMAD5,* and *SMAD9* upon exposure of HUVEC to disturbed flow (*n* = 3). *BMP4* and *SMAD5* showed significant overexpression in venous endothelial cells exposed to disturbed flow compared to parallel uniform flow as early as 24 h of exposure. The expression of *BMP4* and *SMAD5* was significantly higher after exposure to disturbed flow for 48 h. mRNA fold values were calculated relative to static control. All data were normalized with GAPDH expression and are given as relative to static control. (**B**) Disturbed flow at 6 dyn/cm^2^ for 24 h induced a significant expression of BMP4 and pSMAD5 in the venous endothelial cells (scale bar 50 µM, magnification 40×). (**C**) Mean fluorescence intensity was plotted as the average fluorescence intensity ± SD of five fields per flow condition and from three biological replicates. PF indicates parallel uniform shear stress, and DF represents disturbed shear stress without any oscillatory flow. * indicates *p* < 0.05, ** *p* < 0.01, *** *p* < 0.001, and **** *p* < 0.0001 vs. respective static or parallel uniform shear-treated groups.

BMPs belong to the TGFβ superfamily [22], and BMP4-dependent SMAD1/5/9 signaling is known to have a role in vascular calcifications and various cancers [23,24]. The mRNA levels of BMP4 and SMAD1/5/9 were elevated in HUVECs exposed to disturbed flow (Figure 5A). BMP4 and SMAD5 were significantly upregulated at 24 h, unlike TGFβ2/3 isoforms. BMP4 expression was significantly higher (mean 6.443 ± 0.16 folds) compared to TGFβ1 (4.669 ± 0.14) in cells exposed to disturbed flow (*p* < 0.05). Immunofluorescence imaging (Figure 5B,C) revealed a higher cytoplasmic expression of BMP4 and nuclear translocation of pSMAD5 in cells exposed to disturbed flow (*p* < 0.0001 vs. static vs. parallel flow). There was only a minimal expression of BMP4 and pSMAD5 in cells exposed to uniform venous shear stress. Taken together, these observations suggest that EndMT in venous endothelial cells exposed to disturbed flow is mainly dependent on BMP4/pSMAD5-SNAI1/2 signaling.

### 3.6. LDN193189 Abrogates SNAI1/2 Expression Due to Disturbed Flow

We used selective small molecule inhibitors of TGFβ1-SMAD2 and BMP4-SMAD5 pathways to analyze their efficacy to prevent EndMT in venous endothelial cells exposed to disturbed flow. SMAD2/3 inhibitor SB505124, specific for TGFβ type1 and LDN193189 that inhibit BMP4-mediated SMAD1/5/9 signaling, were used in conjunction with disturbed flow conditions.

**Figure 6:**
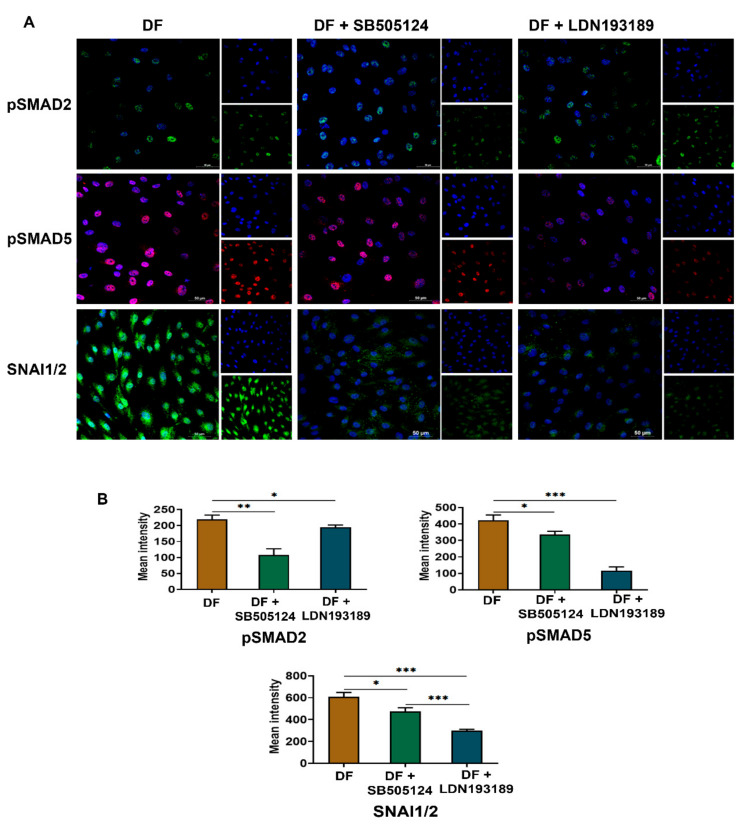
Small molecular inhibitors of SMAD1/5/9 modulate disturbed shear-induced EndMT in venous endothelial cells. (**A**) SMAD2/3 inhibition by 500 nM SB505124 prevented the overexpression of pSMAD2 and pSMAD5 by HUVEC exposed to disturbed flow for 24 h. SNAI1/2 expression due to exposure to disturbed flow was significantly reduced in the presence of SMAD2/3 inhibitor SB505124 (*p* < 0.05). SMAD5 inhibitor LDN193189 reduced the overexpression of pSMAD5 as well as pSMAD2. SNAI1/2 expression was significantly reduced in the presence of LDN193189 (*p* < 0.001) (scale bar 50 µM, magnification 40×). (**B**) Mean fluorescence intensity analysis of five random microscopic fields showed that there was a significant downregulation of SNAI1/2 in the presence of LDN193189 compared to SB505124 (*p* < 0.001). DF represents disturbed shear stress without any oscillatory flow. * represents *p* < 0.05, ** *p* < 0.01, and *** *p* < 0.001 vs. respective static or parallel uniform shear-treated groups.

A concentration of 500 nM and 1 µM was selected for SB505124 and LDN193189, respectively, based on MTT assay (Supplementary Figure S4). The expression of SNAI1/2 was considered as the yardstick to assess the extent of EndMT in the inhibitor assay. BMP pathway inhibitor LDN193189 inhibited pSMAD5 (*p* < 0.001) and pSMAD2 (*p* < 0.05) effectively (Figure 6A,B). SB505124 inhibited pSMAD2 (*p* < 0.05) and and pSMAD2 (*p* < 0.01). Both inhibitors reduced the nuclear translocation of SNAI1/2. LDN193189 is more efficient than SB505124 in decreasingSNAI1/2 expression in cells exposed to disturbed flow. Interestingly, there was a significant reduction in TGFβ1 and BMP4 in the presence of SB505124 and LDN193189 (Supplementary Figure S5).

## 4. Discussion

Our study provides evidence for endothelial to mesenchymal transition in varicose veins collected from patients with chronic venous disease (C3) and in human umbilical vein endothelial cells (HUVEC) subjected to disturbed shear stress in vitro. EndMT-associated markers were expressed in neointimal hyperplastic regions of human varicose veins. When venous endothelial cells cultured in microfluidic chambers were exposed to different venous flow regimes, induction of EndMT was seen augmented even with minimal flow disturbances. We found that bone morphogenetic protein 4 (BMP4)-pSMAD5 signaling has a key role in regulating EndMT in varicose veins and venous endothelial cells exposed to disturbed flow. BMP4-pSMAD5 signaling seems to be an early and prominent event in venous endothelial cells exposed to disturbed flow. Inhibition of BMP4/pSMAD5 activity prevented EndMT in cells exposed to disturbed flow. Our observations also indicate the regulation of EndMT in varicose veins by TGFβ1-BMP4 through a pSMAD-dependent pathway. These findings advance our understanding of the effect of hemodynamic shear stress in low-pressure venous systems and the pathogenesis of varicose veins.

In our earlier studies in varicose veins, we had observed arterialization of the saphenous vein [18]. The proliferation of SMCs associated with higher expression of vimentin and Notch ligands was found in varicose veins. The present study revealed a significantly increased expression of EndMT-related mesenchymal markers and transcriptional factors in the neointima and media of varicose veins; their presence was minimal in healthy veins. To our knowledge, the role of the EndMT in human chronic venous diseases has not been probed earlier.

Given the hemodynamic alterations in venous flow in varicose veins, we studied the effects of laminar and disturbed venous flow using microfluidic shear stress in vitro models and cultured HUVECs. Venous endothelial cells exposed to disturbed flow despite the absence of oscillatory shear stress expressed mesenchymal and EndMT markers. This expression pattern seems to be a pathologic cellular response. The Y-shaped slide used in our study only creates a bifurcation in the flow direction, which results in a non-uniform flow. EndMT markers are expressed by cells exposed to such a minimal flow disturbance. Notably, expressions of these markers in cells exposed to parallel uniform fluid flow with low shear stress were minimal.

Molecular signaling and drug target-based studies in venous diseases, especially varicose veins, are limited. Earlier studies related to EndMT and hemodynamic effects are related to diseases of vein grafts [25]. Vein grafts exposed to arterial flow are known to have increased TGFβ expression within a few hours of exposure to increased shear stress [26].

EndMT phenotype switch during vascular remodeling is induced by various signaling pathways such as TGFβ, Notch, Wnt, etc. [25,26,27,28,29]. Our study focused on the role of EndMT-mediators such as TGFβ 1-3 and BMP4 pathways. We observed a higher expression of *TGFβ1* and *BMP4* and their effector *SMADs* in varicose veins compared to healthy veins. Several other investigators have observed a higher *TGFβ1* expression in varicose veins [30,31,32]. In venous endothelial cells exposed to conditions similar to venous flow, TGFβ1 and BMP4 were the only genes from the TGFβ family, which were overexpressed in the initial 24 h of exposure to disturbed flow exposure. In varicose vein tissues, there was a noticeable expression of TGFβ3. TGFβ3 isoform can be a product of circumferential stretch [33], inflammation [34], and matrix stiffness [35] found in advanced stages of venous insufficiency.

In comparison to TGFβ1 and SMAD2, the expressions of BMP4 and its effectors SMAD5/9 were significantly higher in HUVECs exposed to disturbed flow, especially at the earliest time point we studied. Previous studies have reported that BMP pathway members BMP2 and 4 are highly regulated by blood flow. Uemura et al. found that circulation-dependent mechanosensors such as Yap/Taz induce BMP4 expression in endothelial cells [36]. Several others have shown that BMP4 is a mechanosensitive protein that has a critical role in vascular biology [37,38]. In mouse aortic endothelial cells, BMP4 is expressed in response to oscillatory flow [37,39]. Interestingly, our studies reveal that oscillatory stress is not necessary for the upregulation of BMP4 in venous endothelial cells exposed to disturbed flow. BMP4 /pSMAD5 signaling seems to be activated early in cells exposed to minimally disturbed flow.

The role of BMP4 and its receptor ALK3 and effector SMAD5 in controlling venous identity during embryogenesis is well recognized [40]. Altered TGFβ and BMP signaling result in aberrant vein formation in mice and zebrafish. This study demonstrates that altered expression of BMP4 and TGFβ1 occurs in varicose veins in adult humans as well.

In vitro studies suggest that small molecule inhibitors of the BMP4 receptor can prevent the expression of SNAI1/2 in venous endothelial cells exposed to disturbed flow. The effect of the BMP4 inhibitor was pronounced than that of the TGFβ1 receptor inhibitor. Targeting BMP4/pSMAD5 using small molecule inhibitors as a possible strategy to regress arterialization of varicose veins needs further scrutiny.

We also found a reduced expression of TGFβ1 and BMP4 ligands in response to disturbed flow when cells were treated with SB505124 and LDN193189. This observation correlated with an earlier finding by Walshe et al. in 2013, where they found a reduced TGFβ expression in the presence of TGFβ receptor 1 (ALK5) inhibitor SB525334 [41]. TGFβ-BMP4 pathway comprises numerous ligands, receptors, and their effectors, which are interrelated with several other angiogenesis pathways. For instance, BMP4 is a transcriptional target of the Wnt-β catenin pathway [42]. Zhou et al. have recently shown that LDN193189 inhibits BMP/catenin pathways [43]. We expect similar mechanisms are responsible for the effect on ligand expression by ALK inhibitors.

In summary, our investigations reveal that neointimal hyperplasia during venous wall remodeling in varicose veins is possibly a result of EndMT. Disturbed flow, even without any oscillatory shear stress, can induce EndMT and endothelial dysfunction. BMP4-pSMAD5 signaling is an important mediator of EndMT in varicose veins. BMP4-pSMAD5 could emerge as a target for small molecule therapy in patients with varicose veins.

## Data Availability

All data sets generated and analyzed to support the conclusion of this study is included in this published article and its supplementary files.

## Supplementary Materials

The following are available online at https://www.mdpi.com/article/10.3390/cells10123563/s1. Figure S1: Diagram explaining the principle of flow and shear stress generation in μ- slides; Figure S2: Microscopic photos showing the histological structure of vein (H&E staining); Figure S3: Characterization of HUVEC with von Willebrand factor and Dil acetylated LDL; Figure S4: Standardization of drug concentration for the MTT reduction assay; Figure S5: Small molecular inhibitors of SMAD pathways modulate the expression of TGFβ1 and BMP4 ligands in venous endothelial cells in response to disturbed flow; Table S1: Primers used for real-time PCR; Table S2: Summary of source, dilutions of markers used for Western blot, immunohistochemistry, and immunofluorescence.

## Abbreviations

ALK	Activin receptor-like kinase
BMP	Bone morphogenic protein
Dil-Ac-LDL	Dil-labeled acetylated low-density lipoprotein
EndMT	Endothelial mesenchymal transition
HUVEC	Human umbilical vein endothelial cells
IBIDI	Integrated bio diagnostics
pSMAD	Phospho SMAD
SMAD	Mothers against decapentaplegic homolog 3
SMC	Smooth muscle cells
TGFβ	Transforming growth factor β
vWF	von Willebrand factor
