# Supplementary material for: Shear Stress Alterations Activate BMP4/pSMAD5 Signaling and Induce Endothelial Mesenchymal Transition in Varicose Veins
Source: Cells. 2021 Dec 17;10(12):3563. doi: 10.3390/cells10123563 (PMC8700678; doi:10.3390/cells10123563)
Supplement: Supplementary file 1 [file cells-10-03563-s001.zip › cells-1476825-supplementary.pdf]

Supplementary Materials

Shear stress alterations activate BMP4/pSMAD5 signaling and induce endothelial mesenchymal transition in varicose veins

Karthika CL[1], Ahalya S[1], Vyshna Beena[1], BinilRaj SS[2], RaviKumar B Lakkappa[3],Ravi Kalyani[4], Radhakrishnan N[5], Kalpana SR[4], Kartha CC[6], Sumi S[1]

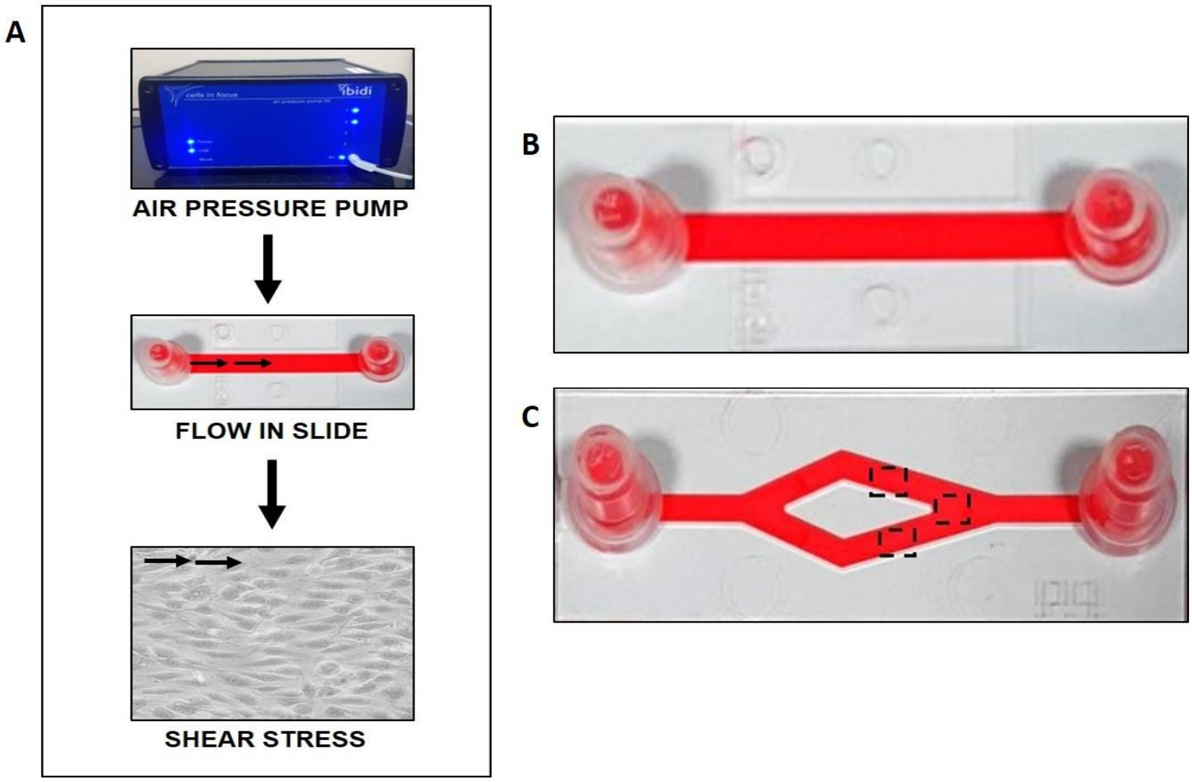

Supplementary Figure S1. (**A**) **Diagram explaining the principle of flow and shear stress generation in μ- slides.** The pump generates a constant air pressure (mbar) which pumps the media from one reservoir to the other and back through the IBIDI channel slides. The applied pressure results in a specific flow rate (ml/min) that is dependent on the pressure input, the viscosity of the medium, and the flow resistance of the perfusion system. The specific flow rate (ml/min) produces a wall shear stress (dyn/cm^2) to which the HUVEC cells were exposed. (**B**) **Photograph of μ–Slide I 0.4 Luer ibiTreat.** μ–Slide I 0.4 Luer ibiTreat was used to ensure unidirectional laminar flow. It is achieved by perfusing medium through low walled channels and by keeping the flow constant over time for both direction and velocity. (**C**) **Photograph of μ–slide Y-shaped ibiTreat.** This slide ensures non-uniform laminar flow mimicking minimal flow disturbances. Dotted square represents the areas around bifurcation.

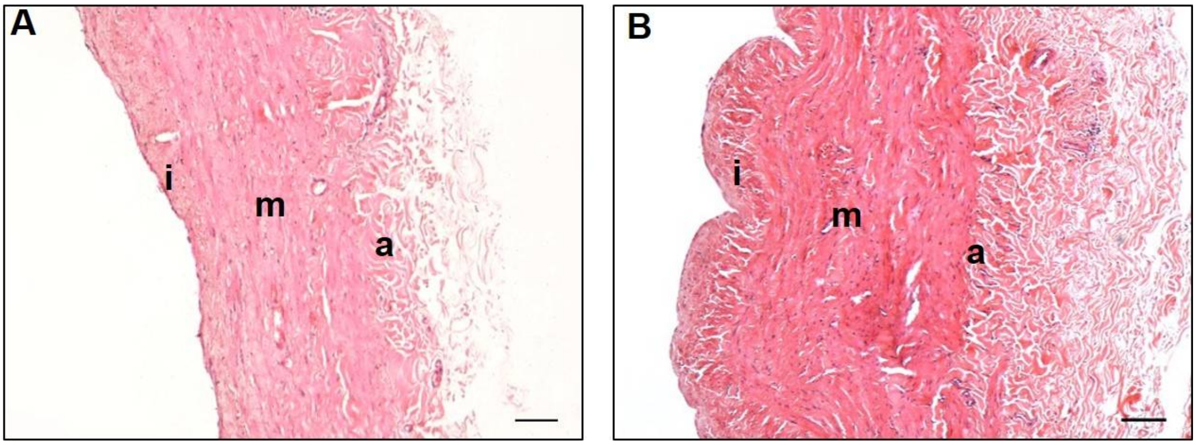

Supplementary Figure S2. Microscopic photos showing the histological structure of vein (H&E staining). (**A**) Control saphenous vein shows the regularity of vein wall with normal thickness of three layers (tunica intima (i), tunica media (m) and tunica adventitia (a). Medial longitudinal bundles of SMCs are visible. (**B**) Section of varicose vein shows the irregular pattern of the wall with thickened neointima (i), VSMC proliferation in media (m) and adventitia (a). Magnification- 10X, Scale bar- 100µm.

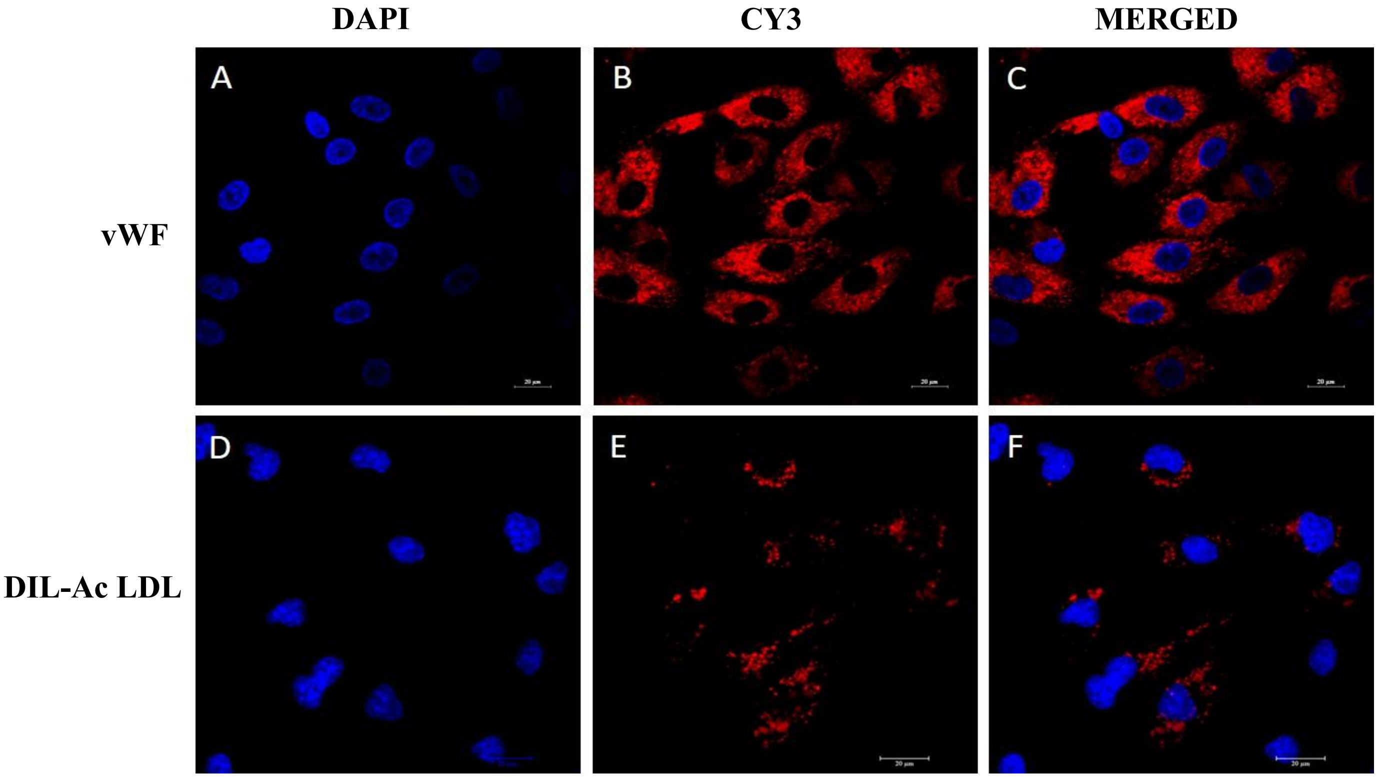

Supplementary Figure S3. Characterization of HUVEC with von Willebrand factor and Dil acetylated LDL. HUVECs exhibit characteristic cobblestone morphology and validated by VWF and Dil acetylated LDL. **Upper panel: von Willebrand factor.** (**A**) Nuclear staining with DAPI (blue), (**B**) anti vWF antibody (1:100, DAKO A0082), (**C**) Merged image. The positive staining obtained with VWF antibody suggested the presence of Weibel-Palade bodies, secretion granules present in endothelial cells. VWF staining was observed in all the cells as punctuate structures. **Lower panel: Dil acetylated LDL.** (**D**) Nuclear staining with DAPI (blue), (**E**) Dil-Ac-LDL (20µg/ml, Life technologies L3484), (**F**) Merged image. Uptake of Dil acetylated LDL showed the phagocytic ability of HUVEC. Magnification-40X, Scale bar- 20µm.

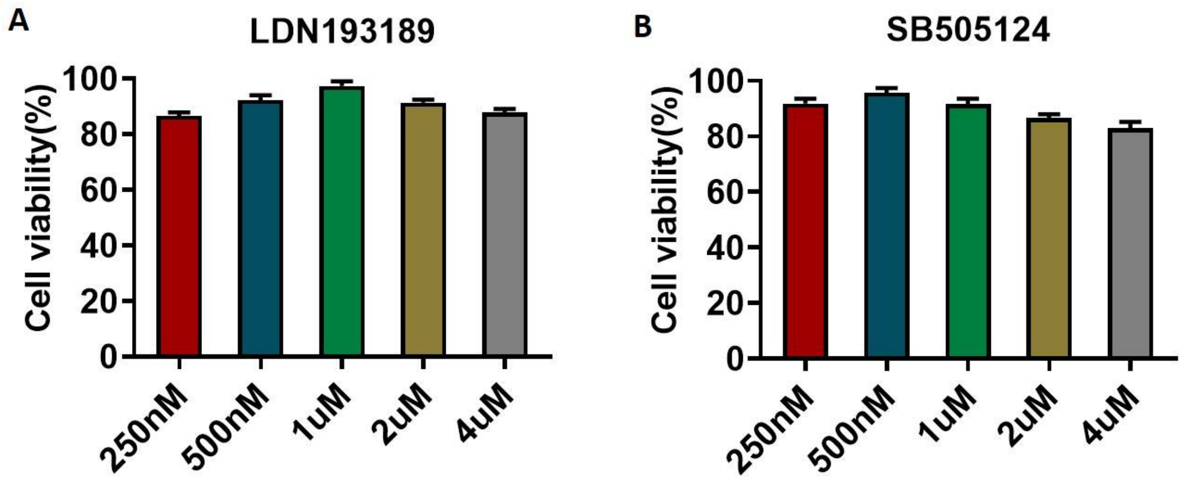

Supplementary Figure S4. Standardization of drug concentration for the MTT reduction assay. (**A**) HUVECs treated with BMP4 pathway inhibitor LDN193189 and(**B**) TGFß1 inhibitor SB505124 for 24 hrs

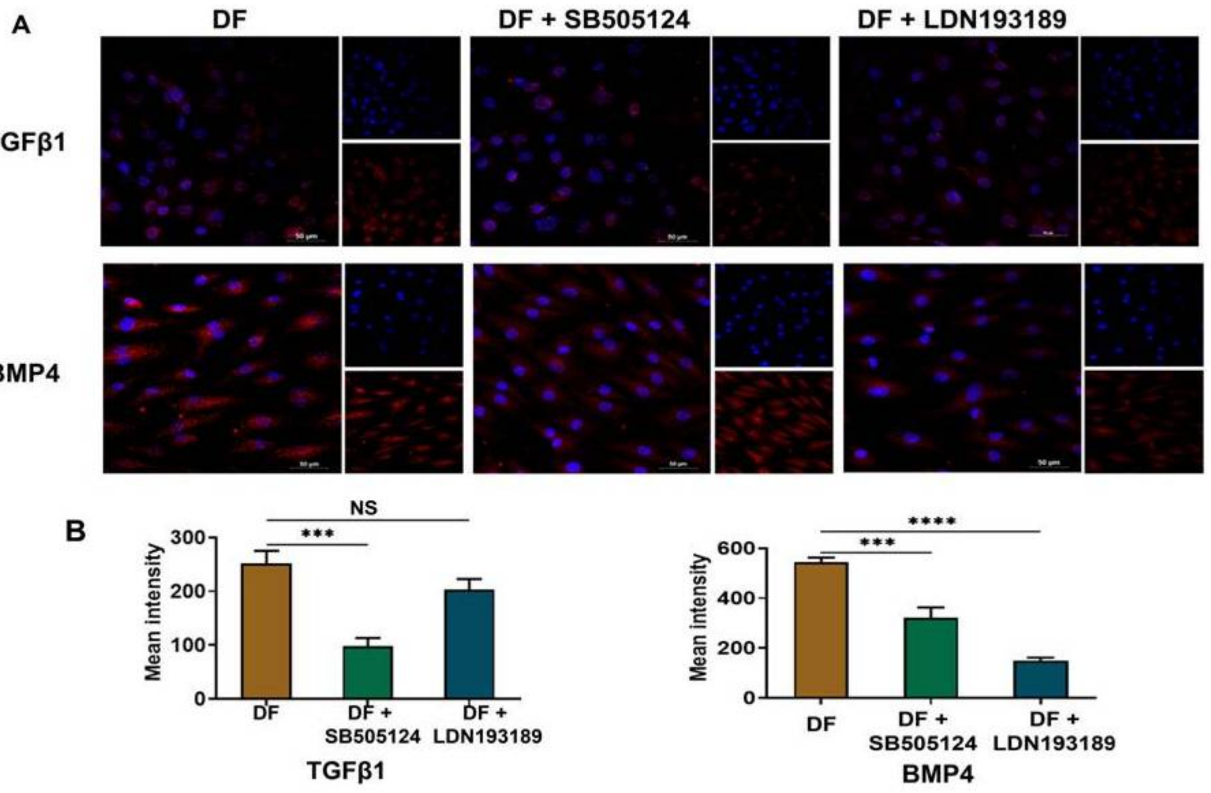

Supplementary Figure S5. Small molecular inhibitors of SMAD pathways modulate the expression of TGFβ1 and BMP4 ligands in venous endothelial cells in response to disturbed flow. (A) SB505124 and LDN193189 prevented the overexpression of TGFβ1 and BMP4 in HUVEC exposed to disturbed flow for 24 hrs. (Scale bar 50μM, magnification 40x). (B) Mean fluorescence intensity analysis of random microscopic fields showed that there was a significant downregulation of TGFβ1 and BMP4 ligands in the presence of ALK inhibitors. DF represents disturbed shear stress without any oscillatory flow. * represents $p < 0.05$, ** $p < 0.01$, *** $p < 0.001$ and **** $p < 0.0001$ *vs* respective static or parallel uniform shear-treated groups.

Supplementary Table S1: Primers used for real-time PCR

Gene	Primer sequence 5'→ 3'	AT (^{0}C)
CD31	F- GACAGTCAGAGTCATTCTTGCCCCA<br>R- CTCGTTGTTGGAGTTCAGAAGTGGT	60
vWF	F- CTGTGACACATGTGAGGAGCCTGA<br>R-TCACTTGCTGCACTTCCTGGG	61
VE- CADHERIN	F- GACCAGGACTTTGACTTGAGCCA<br>R- CGGGGCTGTGGGGTCAGTATC	61
ALPHA- SMA	F- CACAACTGGCATCGTGCTGGAC<br>R- AGTAGTAACGAAGGAATAGCCACGC	60
FIBRONECTIN	F- TGTGACCCTCATGAGGCAACGT<br>R- GAGAATACTGGTTGTAGGACTGGCC	61
TRANSGELIN	F- AATGGCGTGATTCTGAGCAAGCTG<br>R- ATCTCCACGGTAGTGCCCATCATTC	61
CALPONIN1	F- ATGGCGAAGACGAAAGGAAACAAG<br>R- CTGGCTGGCTCCTTTGTTGGTG	61
N- CADHERIN	F- GCAGATAGCCCGGTTTCATTTGAG<br>R- AGGGCATTGGGATCGTCAGCA	63
VIMENTIN	F- TGAACCTGAGGGAAACTAATCTGGA<br>R- ATCGTGATGCTGAGAAGTTTCGTTG	60
SNAI1	F- AATCCAGAGTTTACCTTCCAGCAGC<br>R- AGCCTTTCCCACTGTCCTCATCT	61
SNAI2	F- GAACTCACACGGGGGAGAAGCCT<br>R- GCTACACAGCAGCCAGATTCCTCA	61
TWIST1	F- GAGCAACAGCGAGGAAGAGCC<br>R- TGCGTCTGCAGCTCCTCGTAA	61
TWIST2	F- AGCGGCGCTACAGCAAGAAGT<br>R- CGTCTGGATCTTGCTCAGCTTGT	61
TGFβ1	F- CCAACTATTGCTTCAGCTCCACG<br>R- ACGTAGTACACGATGGGCAGCG	63
TGFβ2	F- CCTCCGAAAATGCCATCCCG<br>R- CAACTTTGCTGTCGATGTAGCGCTG	61
TGFβ3	F- TTCAAAGGCGTGGACAATGAGGAT<br>R- TGCGGAGGTATGGGCAAGGG	61
BMP4	F- CCACGAAGAACATCTGGAGAACATC<br>R- GTCCAGTAGTCGTGTGATGAGGTGC	61
SMAD1	F- CACAAACATGATGGCGCCT<br>R-CATAGTAGACAATAGAGCACCAGTGTTT	61
SMAD2	F- ATAGGTGGGGAAGTTTTTGCTGAGT<br>R- TGCCTTCGGTATTCTGCTCCCC	61
SMAD3	F- GCTGACACGGAGACACATCGGAA<br>R- GCAGCGAACTCCTGGTTGTTGAAGA	61
SMAD4	F- TCCCATTTCCAATCATCCTGCTC<br>R- TCACTAAGGCACCTGACCCAAACA	61
SMAD5	F- TTGGTGGAGAGGTGTATGCGGAA<br>R- CCCAACCCTTGACAAAACTCATCCG	61
SMAD9	F- GGAAAGGGTGTGCACTTGTACTACG<br>R- TCAAAGCCGTGGTGAACTGACTGG	61
GAPDH	F- CCAGGCGCCCAATACGACCAA<br>R- TTCTTTTGCGTCGCCAGCCGA	60

AT denotes annealing temperature, F forward, R reverse

Supplementary Table S2: Summary of source, dilutions of antibodies used for western blot, immunohistochemistry (IHC) and immunofluorescence (IF) assays

Antibody	Source	Manufacturer, Cat. No.	Western blot dilution	IHC dilution	IF dilution
CD31	Rabbit	Novus Biologicals, NB100-2284	1:1000	1:100	1:100
VE- CADHERIN	Rabbit	Abcam, ab33168	-	1:400	-
ALPHA- SMA	Rabbit	Abcam, ab5694	-	1:2000	1:100
FIBRONECTIN	Rabbit	Novus Biologicals, NBP1-91258	-	1:200	-
TRANSGELIN	Mouse	Sigma- Aldrich, MABT167	1:1000	1:400	-
CALPONIN1	Rabbit	Abcam, ab46794	1:1000	1:500	-
SNAI1/2	Rabbit	Abcam, ab180714	1:1000	1:50	1:100
TWIST1	Rabbit	Abcam, ab50581	1:1000	1:100	-
TGFβ1	Rabbit	Abcam, ab92486	1:1000	1:100	1:100
pSMAD2	Rabbit	Novus Biologicals, NBP2-66797	1:1000	1:50	1:100
BMP4	Rabbit	Abcam, ab39973	1:1000	1:100	1:100
pSMAD5	Rabbit	Novus Biologicals, NBP2-67394	1:1000	1:50	1:100
GAPDH	Mouse	SantaCruz, Sc-32233	1:500	-	-
vWF	Rabbit	Dako, A0082	-	-	1: 100
Goat Anti-Rabbit IgG H&L (HRP)	Goat	Abcam, ab97051	1:5000	1:100-1:800	-
Rabbit anti- mouse IgG H and L HRP	Rabbit	Abcam, ab97046	1:5000	1:800	-
Goat polyclonal Secondary Antibody to Rabbit IgG - H&L (Alexa Fluor® 488)	Goat	Abcam, ab150077	-	-	1:200
Goat Anti-Rabbit IgG H&L (Cy3 ®) preadsorbed	Goat	Abcam, ab6939	-	-	1:200
